# Virological failure in a pediatric cohort on a dolutegravir based regimen: a retrospective study in northwest Ethiopia, 2017–2023

**DOI:** 10.3389/fped.2025.1442215

**Published:** 2025-11-17

**Authors:** Woretaw Sisay Zewdu, Mulugeta Molla Zeleke, Yared Andargie Ferede, Achenef Bogale Kassie, Muluken Adela Alemu, Tilaye Arega Moges

**Affiliations:** 1 Department of Pharmacology and Toxicology, School of Pharmacy, College of Health Sciences, Debre Tabor University, Debre Tabor, Ethiopia; 2 Department of Clinical Pharmacy, School of Pharmacy, College of Health Sciences, Debre Tabor University, Debre Tabor, Ethiopia

**Keywords:** virological failure, dolutegravir, antiretroviral therapy, children, HIV/AIDS

## Abstract

**Introduction:**

Despite the fact that antiretroviral therapy (ART) has reduced HIV/AIDS-related morbidity and mortality, pediatrics treatment failure remains a global concern. As a result, this study set out prudently to determine the prevalence of virologic failure and its predictors among children and adolescents on a Dolutegravir (DTG)-based antiretroviral regimen.

**Methods:**

A hospital-based retrospective cross-sectional study was conducted on children and adolescents on ART at Debre Tabor Comprehensive Specialized Hospital in Northwest Ethiopia from February-2017 to September-2023. Study participants were selected purposively. Data was collected using a semi-structured questionnaire and a data abstraction tool. Bivariate and multivariate logistic regression analyses were fitted to determine the linked factors. A *p*-value less than 0.05 was deemed to indicate a statistically significant association.

**Results:**

Among the 359 children and adolescents included in this study, 38 (10.58%) had developed virological failure. The odds of virological failure among children and adolescents were found to be increased by the age of the child <10 years (AOR = 4.41; 95% CI: 2.60–7.47), the care taker being a guardian or neighbor of patient (AOR = 2.03; 95% CI: 1.15–4.73), both parents passing away (AOR = 1.29; 95% CI: 0.12–2.68), CD4 counts ≤200 cells/µL (AOR = 4.3; 95% CI: 1.32–5.9), being infected with OIs (AOR = 2.03; 95% CI: 1.38–3.55), poor adherence status (AOR = 1.37: 95% CI: 1.12–3.11), adverse drug reaction (AOR = 1.75: 95% CI: 1.02–4.97), and anemic (AOR = 1.70: 95% CI: 1.03–5.15.04).

**Conclusion:**

Despite potent DTG-based ARTs being introduced, virologic failure remains a concern in the study area. Special consideration should be directed towards children under the age of 10 years who are in the care of a guardian or neighbors, have lost both parents, are infected with opportunistic infections, have a poor adherence status, are experiencing adverse drug reactions, and anemic.

## 1 Introduction

### 1.1 Background

Public health is significantly jeopardized by the human immunodeficiency virus (HIV). Since the beginning of the epidemic, 85.6 million individuals have contracted HIV, and 40.4 million have died as a result of Acquired immunodeficiency syndrome (AIDS)-related complications (1). Tragically, 75,000 [50,000–110,000] children died from AIDS-related illnesses in 2024, out of 1.4 million [1.1 million–1.8 million] children (0–14 years) living with HIV (1).

Nearly two-thirds (>60%) of the world's HIV-positive population resides in Sub-Saharan Africa, making this continent the worst hit (2). Children in sub-Saharan Africa accounted for nearly 90% of all AIDS-related fatalities (3). As of 2024, the death rate from AIDS among Ethiopia's children (0–14 years) has reached 1,200 [610–2,400] (4).

Joint United Nations Programme on HIV/AIDS (UNAIDS) and world health organization (WHO) launched joint calls to action, urging all nations and international institutions to do what is necessary to halt the HIV/AIDS epidemic and increase access to antiretroviral therapy (ART) (5). The UNAIDS' 95-95-95 aim is to be met by 2030 with the following outcomes: 95% of people living with HIV will know their status, 95% will get antiretroviral medication without interruption, and 95% will maintain a suppressed viral burden while on ART (6). In 2024, globally, meeting the 95-95-95 objectives for children (0–14 years) is difficult, with just 63% [46%–84%] aware of their HIV status, 87% [63%–98%] receiving ART, and 86% [62%–98%] attaining viral suppression (1).

In 2024, 94%, 88%, and 86% of people living with HIV in Ethiopia were cognizant of their HIV status, receiving ART, and successfully suppressing the viral load, respectively, implying that, a significant number of patients' viral loads were not suppressed, which could lead to treatment failure (4).

In 2019, Ethiopia implemented dolutegravir (DTG) as a first line treatment for children and adults on ART. DTG is now used as a component of first-, second-, and third-line treatments. The DTG therapy exhibits robust antiviral efficacy, a substantial resistance threshold (7), and a commendable safety record (8). Despite improvements in the coverage of ART in resource-limited countries, the issue of ART failure continues to be the foremost concern (9). Achieving virologic suppression is the key indicator of an effective HIV/AIDS treatment outcome, citing its high sensitivity and effectiveness (10). In spite of the fact that viral suppression remains the main goal of ART, near to two million and twenty thousand patients globally and in Ethiopia had virological failure, respectively, in 2024 (1, 4).

Diversified factors predict virologic failure. Virologic suppression is less robust in children than in adults due to non-adherence, nevirapine (NVP)-specific problems, formulary constraints, and psychosocial considerations (11). Thus, virologic failure leaves children at risk of poor growth, neurodevelopment, clinical progression, and death (7). Therefore, virologic suppression failure detection and control are crucial in HIV/AIDS care continuum. Continuing to use a failing treatment regimen for an extended period of time raises the likelihood of developing resistance to the drugs (12). To achieve viral suppression, it is critical to accurately detect, understand, and treat predictors of virological failure.

It is extremely difficult to keep viral suppression going in ART-treated children and adolescents over the long term due to poor adherence. Additionally, children and adolescents who do not receive appropriate ART have rapid progression of HIV (13). On top of that, the number of children receiving second-line regimens is low when compared to adults, yet the prevalence of virologic failure is on the rise (14). This implies that patients who do not respond to first-line ART are not being properly identified and are consequently not receiving the necessary modification to their treatment (15). Understanding the current prevalence and factors linked to virological treatment failure is crucial to devising effective strategies and policies.

Despite Integrase Strand Transfer Inhibitors (InSTIs)-based ART regimens being associated with high rates of viral suppression, patients who are at risk for virologic failure while on these regimens must be identified early on to optimize treatment outcomes and prevent the development of drug resistance (16). After the adoption of DTG in 2019 in Ethiopia for children and adolescents, the rate of viral suppression and related parameters has not been predicted in our context. With that in mind, the objective of this research is to examine the prevalence of virological non-suppression and its linking factors in children and adolescents living with HIV receiving DTG-based ART at Debre Tabor Comprehensive Specialized Hospital.

## 2 Methods

### 2.1 Study area

**Figure 1:**
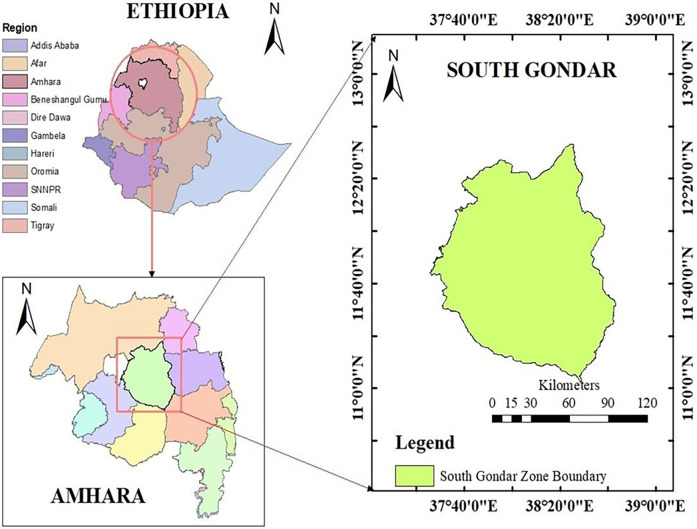
Study area map (ArcMap version 10.4).

This study was conducted at Debre Tabor Comprehensive Specialized Hospital, Northwest Ethiopia. The hospital is situated in the South Gondar Zone, in the Amhara regional state (Figure 1). It is roughly 666 kilometers from Ethiopia's capital, Addis Ababa. Moreover, it is situated at 11˚51′N38˚1′E, 2,706 meters above sea level. Over five million individuals receive inpatient and outpatient services from the hospital. Since the hospital's ART clinic opened its doors in 2005, 2,430 patients living with HIV have begun treatment.

### 2.2 Study design and period

A descriptive cross-sectional study was conducted in northwest Ethiopia from February 2017 to September 2023. The study aimed to determine predictors of virological failure among children and adolescents living with HIV who were receiving first-line DTG-based ART.

### 2.3 Study participants

All children (<10 years) and adolescents (10–19 years) living with HIV who had been on DTG-based ART for more than 6 months were included in the study. Those who have died, transferred out, lost to follow-up, and had incomplete charts with a major variable (viral load) were excluded from this study.

### 2.4 Sample size determination

The sample size was determined using a formula for a single population proportion, with a level of trust of 95%. The proportion of virologic failure on dolutegravir is assumed to be 50%, as no previous study has been conducted on this specific population. A relative precision of 5% is taken into account. The computed size of sample was 384.

$$n=\frac{\left(Z\alpha /2)*P(1-P\right)}{d^{2}}$$ (1)

Since the total number of patients living with HIV on the DTG based regimen is less than 10,000, the sample size is reduced using a correction formula mentioned below (17).

$$\mathrm{Nf}=\frac{\mathrm{no}}{1+\mathrm{no}/N}$$ (2)

Where nf = final sample size, no = calculated sample size (384), and *N* = total population (2,151) nf ≈ 326. To account for non-response, a 10% adjustment was made to the initial sample size of 326, resulting in a final sample size of 359 for this study. The study participants were selected from patients living with HIV who visited the ART clinic during the data collection period using purposive sampling technique.

### 2.5 Study variables

The incidence of virological failure served as the response variable, while gender, age, domicile, disclosure status, age of caretaker, relationship with caretaker, occupation, marital status of the caretakers, characteristics of adherence, history of antiretroviral (ARV) prophylaxis use as a prevention of HIV transmission from mother to child, ART regimen type, isoniazid prophylaxis, cotrimoxazole prophylaxis, nutritional status, WHO clinical stage, CD4 count, and recent opportunistic infection are considered control variables.

#### 2.5.1 Definition of variables

Virological failure was defined as a viral load over 1,000 copies/mL, confirmed by two consecutive measurements, occurring 6 months post-initiation of antiretroviral therapy (ART), and following 3 months of enhanced adherence counseling after the initial viral load assessment (18).

Enhanced Adherence Counseling (EAC): in the Ethiopian context it is systematically conducted over three key sessions for patients with uncontrolled viral load (VL). The process is documented, and interventions are provided monthly, with VL re-monitoring 3 months after effective EAC. The EAC continues for 3–6 months after regimen changes. In the first session of EAC, barriers to adherence whether cognitive, behavioral, or socio-economic are addressed, along with risk reduction, motivation, and a mental health screening. In the second session, the initial adherence plan is reviewed and adjusted based on challenges faced, and adherence is assessed through a pill count. Finally, by the third session, adherence is reassessed, the plan is further refined, and a decision is made on whether to repeat VL testing or continue counseling. This structured approach sets patients on the right path toward better health outcomes (19).

Adherence to ART medications was categorized as good, fair, or poor based on the calculated percentage of missed drug doses relative to the total monthly dose, as per established criteria shown below (18) (Table 1).

Anemia: was defined according to WHO criteria (hemoglobin <11 g/dL for children <5 years; hemoglobin <11.5 g/dL for children 5–11.99 years; hemoglobin <12 g/dL for children 12–14.99 years; hemoglobin <12 g/dL for females aged ≥15 years; hemoglobin <13 g/dL for males aged ≥15 years) (20).

CD4 count: categorized as per the WHO, is the appropriate classification to describe their immunological level. Children under age 1 and who had a CD4 cell count <1,500 cells/mm^3^; children aged between 1 and 3 years and who had a CD4 cell count <750 cells/mm^3^; children aged between 3 and 5 years and who had a CD4 cell count <350 cells/mm^3^; and children aged between 5 and 15 years and who had a CD4 cell count <200 cells/mm^3^ will be categorized as having a CD4 cell count below threshold (21).

Adverse drug reaction: A response that is appreciably noxious and unintended, and which occurs from an intervention related to the use of a medicinal product at doses normally used in humans for the prophylaxis, diagnosis, or therapy of disease, or for the modification of physiological function (22).

### 2.6 Data collection tool and procedure

Data was gathered by utilizing a suitable data extraction tool that has been modified to operate in the English language and is adapted from the Ethiopian Federal Ministry of Health ART clinic intake and follow up form (Supplementary Material S1). It was employed to extract information from the national HIV intake and follow-up care records, after ensuring that the necessary variables were present in the patients living with HIV registration book. Furthermore, the interviewer administered a semistructured questionnaire that had been developed by consulting different literature in the English language, which had been translated in to Amharic and then again translated into English. To affirm their consistency, comparisons were done between the two versions. The questionnaire underwent additional modifications following a pretest conducted on a 5% subset of the study group at Debre Tabor Health Center. Three health professionals (a physician, a pharmacist, and a nurse) who have experience working in ART clinics were selected for data extraction. In addition, they were trained for 1 day in the hospital before the start of data collection. Upon using the patient's registration number from the database, the patient's charts were retrieved. Finally, charts that had a completion date for ART enrollment and a date for viral load measurement were selected, and variables were also documented. Data were extracted from April 1 to April 15, 2024, from patient charts that fulfilled the inclusion criteria. The data retrieval process is closely monitored by the principal investigator and two supervisors.

### 2.7 Data process and analysis

The data was entered into Microsoft Excel and exported to Stata version 17 software (STATA corp., College Station, TX, USA) for analysis. A binary logistic regression model was used to identify linking factors with virologic failure. Multivariate binary logistic regression was used to control the impact of confounders for variables with *p*-values of ≤0.2 in the bivariate binary logistic regression analysis. Factors were deemed significantly linked to the response variable if their *p*-values were ≤0.05.

### 2.8 Ethical consideration

The Institutional Research Ethical Review Committee (IRERC) of Debre Tabor University granted ethical approval under reference No DTU/Res/305/16.

Afterwards, correspondence was sent from Debre Tabor University to the relevant authorities at Debre Tabor Comprehensive Specialized Hospital. Then the hospital officials allowed us to conduct this research. Each participant or caretaker in the study gave their verbal informed consent after getting the study's purpose stated to them before the interview. The respondent's privacy was protected, and all data obtained was de-identified.

## 3 Results

### 3.1 Sociodemographic characteristics

Among the 359 patients living with HIV on DTG-based ART regimens included in this study, 63% were children. Of these, more than half (54.9%) were female, and the mean age was 7.60 ± 0.29 years. A majority of the participants were urban dwellers; 215 (59.9%) had had a primary school education (192, 53.5%) (Table 2).

**TABLE 1:** Assessment of ART treatment adherence status of patients with HIV in Ethiopia, 2024.

Adherence status	Percent (%)	Patient's ART regimen as once daily (QD)	Patient's ART regimen as twice daily (BID)
Good	>95%	<2 doses missed per month	<3 doses missed per month
Fair	85%–94%	3–4 doses missed per month	4–9 doses missed per month
Poor	<85%	>5 doses missed per month	>9 dose missed per month

**Table 2:** Socio-demographic characteristics of children and adolescents on DTG-based ART and their caretaker in Debre Tabor comprehensive specialized hospital, 2017–2023 (*N* = 359).

Socio-demographic variable	Categories	Total, *N*	%
Age (years)	<10	226	63.0
	≥10	133	37
Sex	Male	162	45.1
	Female	192	54.9
Domicile	Urban	215	59.9
	Rural	144	40.1
Educational status of child	No formal education	192	53.50
	Primary	126	35.10
	Secondary	41	11.40
Religion	Orthodox	373	93.90
	Muslim	11	3.05
	Other^a^	11	3.05
Educational status of caretaker	Illiterate	92	25.60
	Primary	75	20.90
	Secondary	123	34.30
	Tertiary	69	19.20
Ethnicity	Amhara	307	85.50
	Tigrie	47	13.10
	Others^b^	5	1.40
Marital status of caretaker	Single	70	19.5
	Married	244	68
	Divorced	38	10.6
	Widowed	7	1.90
Occupation of caretaker	Unemployed	176	49
	Employed	183	51
Family size	<4	206	57.4
	≥4	153	42.6
Age of caretaker	<45	142	39.60
	≥45	217	60.40
Parental status	Both alive	84	23.4
	Father alive	67	18.7
	Mother alive	79	22
	Both died	129	35.90
Relationship between child and caretaker	Parent	265	73.8
	Relative	63	17.5
	Guardian/neighbors	31	8.6

a Other, Catholic and Protestant.

b Others, Oromo and Guragie.

### 3.2 Clinical features and laboratory profiles of children and adolescents

Current DTG-based ART regimen includes tenofovir (TDF) + lamivudine (3TC) + DTG, abacavir (ABC) + 3TC + DTG, and zidovudine (AZT) + 3TC + DTG. Virologic failure was detected in 38 participants (10.58%), nearly half (48%) of whom were female. A high proportion of children and adolescents have a history of severe acute malnutrition (162, 45.1%), opportunistic infections (186, 51.8%), ARV prophylaxis for prevention of mother-to-child transmission of HIV (81.60%), were anemic (30.9%), and ART-linked ADR (46.2%). The mean overall duration on DTG-based ART was 44.47 ± 3.06 months; more than two-thirds of the participants (262, 78.4%) had a baseline CD4 count above 200 cells/μL; and a majority of the participants were classified as WHO clinical stage I and II (Table 3).

**Table 3:** Clinical, laboratory, and ART profile of children and adolescents on DTG-based ART and their caretaker in Debre Tabor comprehensive specialized hospital, 2017–2023 (*N* = 359).

Variable	Category	Total, *N*	%
HIV status of caretaker	Positive	128	35.70
	Negative	80	22.30
	Unknown	151	42.10
Disclosure status	Disclosed	221	61.60
	Not disclosed	138	38.40
Severe acute malnutrition	Yes	162	45.10
	No	197	54.90
Previous ART experience	Non naïve	249	69.40
	Naïve	110	30.60
Baseline virological status	Suppressed	195	73.31
	Non-suppressed	54	21.69
Previous ART regimen	AZT + 3TC + NVP	65	18.1
	AZT + 3TC + EFZ	41	11.4
	TDF + 3TC + EFZ	32	8.9
	TDF + 3TC + NVP	44	12.3
	ABC + 3TC + NVP	14	3.90
	ABC + 3TC + LPVr	34	9.50
	TDF + 3TC + LPVr	8	2.20
	AZT + 3TC + LPVr	5	1.40
	ABC + 3TC + EFZ	6	1.70
Current ART regimen	TDF + 3TC + DTG	139	38.70
	ABC + 3TC + DTG	111	30.90
	AZT + 3TC + DTG	109	30.40
Duration on DTG (month)	6–12	122	34.00
	12–48	157	43.70
	>48	80	22.30
PMTCT	No	293	81.60
	Yes	66	18.40
Anemic status	Yes	111	30.90
	No	248	69.10
INH prophylaxis	Yes	299	83.30
	No	60	16.70
Cotrimoxazole prophylaxis	Yes	316	88.30
	No	42	11.70
ADR	Yes	166	46.20
	No	193	53.80
CD4 count	>200 cells/μL	282	78.60
	≤200 cells/μL	77	21.40
WHO Clinical HIV/AIDS stage	Stage-I	187	52.10
	Stage-II	116	32.30
	Stage-III	53	14.80
	Stage-IV	3	8.00
Current virologic status	Detectable (>150 copies/mL)	152	42.30
	Not detectable (<150 copies/mL)	207	57.70
Adherence status	Poor	90	25.10
	Fair	30	8.40
	Good	239	66.60
Current virological suppression status	Suppressed	321	89.42
	Nonsuppressed	38	10.58

ADR, adverse drug reaction; ART, antiretroviral therapy; AZT, zidovudine; INH, isoniazid; 3TC, lamivudine; CD4, cluster of differentiation 4; HIV, human immunodeficiency virus; AIDS, acquired immunodeficiency syndrome; NVP, nevirapine; EFZ, efavirenz; TDF, tenofovir; ABC, abacavir; LPVr, lopinavir combined with ritonavir; PMTCT, prevention of mother to child transmission; WHO, world health organization.

**Figure 2:**
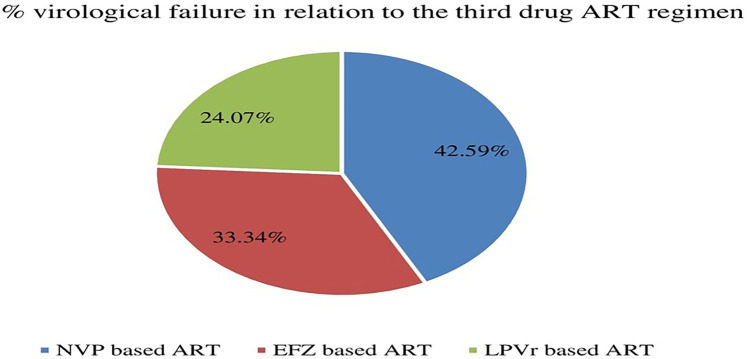
Distribution of virological failure status prior to DTG-based ART initiation in relation to the third drug among children and adolescents. EFZ, efavirenz; NVP, nevirapine; DTG, dolutegravir; LPVr, lopinavir combined with ritonavir.

Before switching to DTG-based ART, virologic failure in those ART experienced patients were 21.69% (54/249). Analysis of third-drug comparisons prior to switching to DTG revealed that NVP-based ART regimens exhibited a significantly greater rate of virologic failure compared to other alternatives (Figure 2).

**Figure 3:**
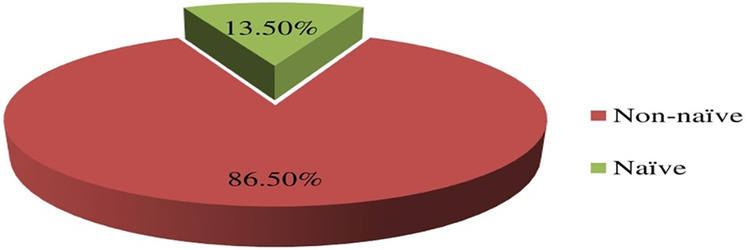
Distribution of failed current virological status in relation to the ART-experience prior initiation of DTG-based ART in children and adolescents. ART, antiretroviral therapy.

A significant proportion of patients with current virologic failure status were ART-experienced, accounting for 86.50% (33/38) of the cases (Figure 3) which underscores the challenges in maintaining viral suppression among non-naïve ART patients.

**Figure 4:**
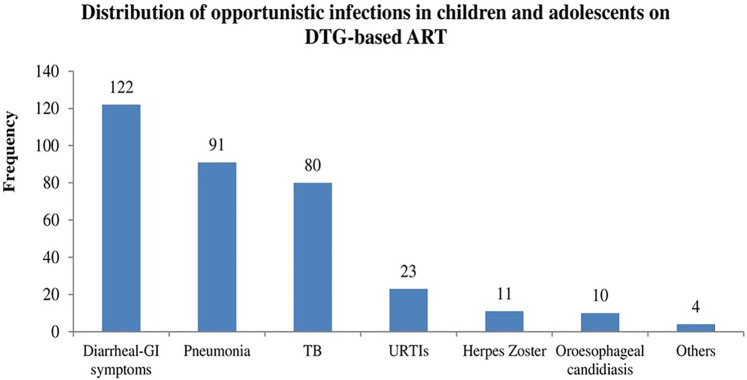
Frequency of opportunistic infections in children and adolescents. DTG, dolutegravir; ART, antiretroviral therapy; GI, gastrointestinal; URTIs, upper respiratory infections; TB, tuberculosis.

Opportunistic infections remain a challenge despite the introduction of InSTIs. Herein, as depicted in Figure 4 below, opportunistic infections are common in children and adolescents, where diarrheal gastrointestinal symptoms, TB and pneumonia take a lion's share.

**Figure 5:**
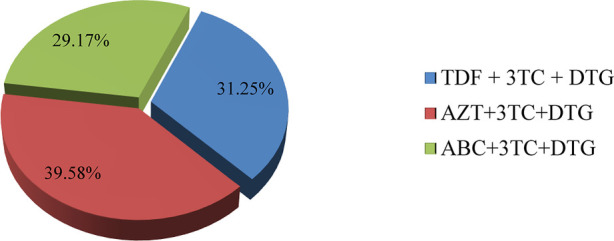
Distribution of failed current virological status in relation to current treatment regimen in children and adolescents. TDF, tenofovir; 3TC, lamivudine; DTG, dolutegravir; AZT, aidovudine; ABC, abacavir.

The distribution of virologic failure in this study was comparable among all DTG-based ART regimens, as shown below (Figure 5).

### 3.3 Factors associated with virological failure among children and adolescents with DTG-based ART

In the bivariate analysis, age of child, relationship of children with caretakers, parental status, ART experience, CD4 count, history of OIs, poor adherence, ADR, and anemia were moderately associated with virologic failure. Explanatory variables including gender, relationship to children, family occupation, HIV status of caretaker, ART types, and anemia with a *p*-value <0.2 in the bivariate analysis become candidate variables for multivariate regression model (Table 4).

**Table 4:** Bivariable and multivariable logistic regression analysis of factors linked with virologic failure among children on DTG-based ART at Debre Tabor comprehensive hospital, north west Ethiopia, 2017–2023 (*n* = 359).

Variables		Bivariate analysis			Multivariate analysis		
		COR	95% CI	*p*-value	AOR	95% CI	*p*-value
Age of child (years)	<10	1.38	[1.11–3.59]	0.006	4.41	[2.60–7.47]	0.01
	≥10	Ref			Ref		
Relationship of children with caregiver	Parent	Ref			Ref		
	Relatives	0.61	[0.19–1.978]	0.41	1.98	[0.99–3.69]	0.96
	Guardian/Neighbors	1.72	[1.15–3.4.3]	<0.001	2.03	[1.15–4.73]	0.03
Parental status	Both alive	Ref			Ref		
	Father alive	0.92	[0.295–2.89]	0.89	1.15	[0.03–8.2]	0.35
	Mother alive	1.71	[0.79–6.06]	0.41	1.71	[0.92–3.06]	0.23
	Both died	3.29	[1.12–10.68]	0.005	1.9	[1.22–2.68]	0.03
ART experience before DTG	Non-naïve	0.28	[0.12–0.69]	0.005	0.78	[0.12–1.98]	0.94
	Naive	Ref			Ref		
CD4 count	>200 cells/µL	Ref			Ref		
	≤200 cells/µL	4.3	[2.02–11.90]	<0.001	4.3	[1.32–5.90]	0.04
Viral load status prior DTG	Suppressed	Ref					
	Nonsuppressed	1.59	[0.91–4.13]	0.24			
Duration on DTG (month)	6–12	2.24	[0.13–4.79]	0.26			
	12–48	3.85	[0.44–6.35]	0.39			
	>48	Ref					
History of OIs	No	Ref			Ref		
	Yes	9.9	[2.08–15.57)	0.001	2.03	[1.38–3.57)	0.003
Adherence	Good	Ref			Ref		
	Fair	4.85	[0.98–8.98]	0.375	3.93	[0.8–6.37]	0.67
	Poor	4.71	[2.1–10.31]	<0.001	1.37	[1.12–3.11]	0.01
ADR	No	Ref			Ref		
	Yes	2.13	[1.22–5.97]	<0.001	1.75	[1.12–4.97]	0.01
Anemia	No	Ref			Ref		
	Yes	1.87	[1.32–2.15]	<0.001	1.70	[1.23–5.15]	0.02

ADR, adverse drug reaction; AOR, adjusted odds ratio; ART, antiretroviral therapy; CD4, cluster of differentiation 4; DTG, dolutegravir; HIV, human immunodeficiency virus; Ref, reference; OIs, opportunistic infections; COR, crude odds ratio.

In the multivariate logistic regression analysis after adjusting the potential confounders: age of child <10 years (AOR = 4.41; 95% CI: 2.60–7.47; *p*-value = 0.01), care taker being guardian or neighbors (AOR = 2.03; 95% CI: 1.15–4.73; *p*-value = 0.03), both parents passed away (AOR = 1.9; 95% CI: 1.22–2.68; *p*-value = 0.03), CD4 counts ≤200 cells/µL (AOR = 4.3; 95% CI: 1.32–5.9; *p*-vale = 0.04), being infected with OIs (AOR = 2.03; 95% CI: 1.38–3.55; *p*-value = 0.003), poor adherence status (AOR = 1.37: 95% CI: 1.12–3.11; *p*-value = 0.01), ADR (AOR = 1.75: 95% CI: 1.02–4.97; *p*-value = 0.01), and anemia (AOR = 1.70: 95% CI: 1.03–5.15.04; *p*-value = 0.02) were the independent determinant and significantly associated with virological failure (Table 4).

## 4 Discussion

InSTIs are powerful anti-HIV drugs. DTG-based ART is valuable and safe for patients living with HIV (23). As of right now, InSTIs-based ART is the gold standard for treating HIV infections. Despite their effectiveness, first-generation InSTIs have a lower genetic barrier to resistance. But the highly effective and advanced second-generation InSTIs (e.g., DTG) are effective against first-generation InSTIs resistant and sensitive HIV strain (24).

In this study, the response of pediatric patients to DTG-based ART was good (89.42%) while falling short of achieving UNAID's goal of 95% by 2030. The overall virological failure was 10.58% (95% CI: 7.21–14.5). This finding is consistent with previous studies conducted in Ethiopia despite a difference in ART regimen type: Amhara regional state, 7.7% (25), Debre Markos and Felege Hiwot referral hospitals, 12.1% (26), a retrospective cohort study done on pediatrics and adolescents in comprehensive specialized hospitals of East Amhara region of northeast Ethiopia (12.47%) (27), and Felege-Hiwot referral hospital, 10.7% (28). As compared to studies in Ethiopia (18.3%) (29), Addis Ababa (17.2%) (30), Tikur Anbessa Specialized Hospital (22.6%) (31), Hawassa 28.2% (32), rural Cameroon 53% (33), Malawi 66% (34) and Cameroon (22.2%) (35), the current study shows that virologic suppression is moderately better. The discordance could be attributed to the purposive inclusion of children and adolescents in a potent InSTIs-based ART, variations in sample size, study population, study time frame, and the diversity in measures of virological failure. For instance, research conducted by Misasew et al. consisted of a sample size of only 250 participants who were under the age of 15 years (30). In terms of the heterogeneity in determinations of virological failure, Enone et al. conducted a study where they diagnosed virological failure based on a single plasma viral load above 1,000 copies/mL (35). In contrast, our study diagnosed virological failure based on two successive plasma viral load determinations of 1,000 copies/mL and above. Another potential explanation for the disparities could be attributed to the divergence in the study time, as there were modifications in treatment guidelines periodically. This is evident in a study done in Addis Ababa (30) and Cameroon (33), which took place prior to the implementation of InSTIs-based ART.

Virologic failure was impacted by a range of sociodemographic, clinical, and therapeutic related factors. In this study, among sociodemographic factors, being an orphan, child-caretaker not biologically-related, and the age of the child being younger than 10 years were associated with virologic failure.

Being a child (age ≤ 10 years) carries greater odds of developing virologic failure as compared to adolescents (AOR = 4.41; 95% CI: 2.60–7.47; *p*-value = 0.01). This finding is in concordance with other work conducted by Leroy et al. (36), Gelaw et al. (AOR = 2.4, 95% CI: 1.0–5.7) (37), Jobanputra et al. (38). (AOR 2.6, 95% CI 1.5–4.5), Emmett et al. (*p* = 0.02) (39), and Makadzange et al. (40). This phenomenon can be attributed to the fact that children's immune system was underdeveloped and the compounding issue of suboptimal, non-pediatric-friendly ART formularies may lead to poor medication adherence (41). As a result, untreated children have faster disease progression in comparison to untreated adults (42). Moreover, adults, unlike younger children, may have access to HIV/AIDS education in educational institutions, which might help them comply with their therapy. On the contrary, a prospective cohort study conducted among children and adolescents living with HIV in Cameroon by Enone et al. (35) and in Oromia, Ethiopia, by Yassin et al. (43) revealed that being a child safeguards against experiencing virologic failure. This could be explained by the fact that, among younger children, their medication has primarily been administered consistently by caregivers.

Being orphaned and having a poor adherence level are independent predictor covariates of virologic failure in the present study. A meta-analysis on the adherence level of orphanage children and adolescents on ART revealed that a significant portion of (22. 0%, 95% CI: 67.4–87.7; *I*^2^ = 82.92%, *p* < 0.001) the participants were poorly adherent to their treatment (44). The suboptimal adherence rate in the present study (33.5%) was in line with other studies (30.3%) done on pediatric patient groups (<15 years) (45) and the study in Waghimra (46). This finding was also corroborated by a scoping review on determinants of treatment failure, which revealed that insufficient treatment adherence leads to failed therapy (47). ADVANCE and NAMSAL are two historic clinical trials performed solely in sub-Saharan Africa reported that DTG-containing ART regimens such as Emtricitabine (FTC) + TDF + DTG, FTC + tenofovir alafenamide (TAF) + DTG (48) and TDF + 3TC + DTG (49) were deleteriously impacted by imperfect adherence (50). In line with the other studies, nonadherence to therapy has been shown to be one of the most common reasons for treatment failure (51, 52).

The current study found that the severity of immunodeficiency is a key determinant, with a lower CD4 cell count linked to virologic failure for INSTI-based ARTs at greater odds, a finding corroborated by other studies such as umbrella reviews (47, 53). Patients who have a low CD4 count (e.g., <200 cells/μL) have either not been receiving ART or are receiving inappropriate regimens; they inherently possess a larger viral reservoir and a higher risk for virologic failure.

This severe immunosuppression is the factor that determines the patient's risk for OIs. In the current study, the odds of having virologic failure were 2.03 times higher among patients with a history of OIs compared with those without, supporting the idea that the absence of OIs predicts good viral suppression (54). This could be because OIs, which are more common in immunocompromised patients with lower CD4 cell numbers, reduce their already compromised immunity and boost viral replication in thousands of copies. This heightened replication pressure can lead to the emergence of mutant forms that eventually acquire drug resistance and cause failed virologic suppression. Furthermore, patients with OIs often employ a variety of medications outside of ART, which may have triggered unfavorable adverse effects that impacted their adherence (55).

In this work, being anemic and not supervised by biological family were linked with virologic treatment failure. Accordingly, anemic children were nearly two times more likely to have a higher risk of developing virological failure when compared with their non- anemic counterparts. A comparative research conducted in Africa evaluating the viral nonsuppression rate in anemic and non-anemic children (*p* = 0.002) (56) and a study at the University of Gondar Hospital (AOR = 5.50:95% CI: 1.37–22.04) (57) lends credence to the current study finding. The mechanism underlying the inadequate virological response in anemic children remains unknown. It might possibly due to anaemia is a hallmark of more advanced disease. Since advanced disease is itself a major risk factor for virologic failure, the observed association with anemia may be largely due to this. Another possible explanation is that anemia negatively impact a patient's adherence to the complex daily ART regimen, directly increasing the risk of virologic failure (57).

Despite data from clinical studies indicating that dolutegravir-based ART regimens had reduced rates of nonadherence owing to their minimal side effects (48, 58), in real-world clinical settings, ADRs constitute the main deterrent to the efficacy of InSTIs-based ART (AOR = 1.75: 95% CI: 1.02–4.97; *p*-value = 0.01). This study's finding is in line with studies conducted in Waghimra (46), Nigeria (59), and Malaysia (60). Additionally, a retrospective cohort study in Ethiopia and a systematic review by Daltro et al. (61) both found that major ADRs are the primary reasons for poor retention, ART regimen change, morbidity, and fatalities in pediatrics (62). Likewise, numerous studies have shown that people who have had drug toxicity will abandon taking antiretroviral therapy, which can lead to acquired drug resistance and virologic failure (36, 37, 63). Prudent use of InSTIs-based ARTs and unprecedented efforts should be placed to diagnose ADRs early.

Even while InSTIs work, other studies have shown that factors including a high viral load to begin with, the disease's stage according to the WHO, the use of PMTCT prophylaxis, and the family's financial level are still important (47). Nonetheless, in the current study, they remain insignificant; the discrepancy might be due to differences in study population and period (51).

## 5 Conclusion

Despite potent DTG-based ARTs being introduced, virologic failure still remains a concern in the study area. Special consideration should be directed towards children under the age of 10 who are in the care of a guardian or neighbors, have lost both parents, are infected with opportunistic infections, have a poor adherence status, are experiencing adverse drug reactions, and are anemic. A prospective multicenter analytical design with mixed research is recommended for robust evidence synthesis.

## 6 Strength and limitation of the study

Despite our best efforts to estimate virological failure and its predictors, there are certain limitations that we must acknowledge. We have tried to control potential confounders using multivariate logistic regression analysis and by the relatively large sample size of the study participants. The Hosmer-Lemeshow goodness-of-fit test was applied to investigate the model's validity. Drawing firm conclusions about the link between virologic failure and predictive factors from this study is less likely because it was a hospital-based, single-centered retrospective cross-sectional study based on primary and secondary data. Despite its shortcomings, this work can serve as a high-grade ore for policymakers and the scientific community.

## Data Availability

The original contributions presented in the study are included in the article/Supplementary Material, further inquiries can be directed to the corresponding author.
