# Supplementary material for: Virological failure in a pediatric cohort on a dolutegravir based regimen: a retrospective study in northwest Ethiopia, 2017–2023
Source: Front Pediatr. 2025 Nov 17;13:1442215. doi: 10.3389/fped.2025.1442215 (PMC12665690; doi:10.3389/fped.2025.1442215)
Supplement: Supplementary file 1 [file Datasheet1.pdf]

ANNEX: Data Abstraction Tool

A. Participants identification

Code No__________________Hospital________________

Part I. Socio-demographic characteristics of ART experienced children and their family /caretaker:

1. Age in complete years--------------<1 year (months): -------------
2. Relationship of caregivers for the child:
   1. Parent 2. Relatives 3. Guardians/neighbors
3. Age of the caregivers--------------
4. Family size------------------------
5. Monthly income of family in Ethiopian Birr---------------------------
6. Gender 1. Male 2. Female
7. Domicile 1. Urban 2. Rural
8. Religion 1. Orthodox 2. Muslim 3. Protestant 4. Catholic 5. Others
9. Ethnic group 1. Amhara 2. Tigray 3. Oromo 4. Others
10. Marital status of caregivers: 1. Single 2. Married 3. Divorced 4. Widowed 5. Separated
11. Educational status-- 1. No formal education 2. Elementary school

3. Secondary school 4. College and above

12. Occupation of caregivers: 1. Unemployed 2. Government employee 3. Farmer 4. House wife 5. Private employee 6. Daily laborer 7. Nongovernmental Organization employee 8. Self-employed
13. Parental status: 1. Both alive 2. Father alive 3. Mother alive 4. Both deceased 5. Unknown

14. HIV status of caretaker: 1. Positive 2. Negative 3. Not known

15. Number of months living with HIV---------------------------

16. Does anyone else know about your HIV Status (children and adolescents)? 1.Yes, 2. No

17. Family education: 1. No formal education 2. Primary school 3. Secondary school 4. College and above

18. Distance from home to ART clinic: 1. <10 Kilometers 2. >10 Kilometers

Part II. Clinical characteristics:

19. Recent CD4 count level (cells/µL) after using DTG-based regimen: -------------

20. Last virological outcomes before switching to DTG-based regimen: -------------

21. Recent virological outcomes after using DTG based regimen

22. Current WHO Clinical HIV/AIDS stage: 1. Stage I 2. Stage II, 3. Stage III, 4. Stage IV

23. Previous ART experience: 1. Naïve 2. Non-naive

24. If Non-naive mention last ART regimen before switch to DTG-based regimen:----------------

1. 1c, AZT-3TC-NVP 2. 1d, AZT-3TC-EFV 3. 1e, TDF-3TC-EFV 4.1f, TDF-3TC-NVP

25. Mention current ART regimen: 1. TDF+3TC+DTG 2. ABC+3TC+DTG 3. AZT+3TC+DTG 4. DRV/r+DTG+ABC+3TC 5. DRV/r+DTG+AZT+3TC 6. DRV/r+DTG+TDF+3TC

26. Virological Status prior initiation of DTG-based regimen.

1. Suppressed 2. Non-suppressed 3. Naïve

27. Duration on ART (in months): ---------------------------

28. Duration on DTG-ART (in months): ---------------------------

29. Hemoglobin: A. --------------------   B. Not done

30. History of isoniazid prophylaxis: 1. Yes   2. No

31. History of Cotrimoxazole prophylaxis: 1. Yes   2. No

32. Recent history of OIs: 1. Yes   2. No

33. If yes: 1. TB   2. Pneumonia   3. Oral thrush   4. Zoster   5. Diarrhea   6. CNS toxoplasmosis

7. Others mention it-------------------

34. History of sever acute malnutrition status: 1. No   2. Yes

35. Did you experience drug adverse effect/s after switching to DTG-based regimen? 1. Yes   2. No

36. How many ARV dose/s do you take/day? 1. Once   2. Twice

37. Did you miss ARV doses in the past one month?   1. Yes   2. No

38. If yes, how many dose/s did you miss?   ---------------------

39. History of prevention of mother to child transmission (PMTCT) ARV services 1. Yes   2. No
