# Supplementary material for: Virological failure in a pediatric cohort on a dolutegravir based regimen: a retrospective study in northwest Ethiopia, 2017–2023
Source: Front Pediatr. 2025 Nov 17;13:1442215. doi: 10.3389/fped.2025.1442215 (PMC12665690; doi:10.3389/fped.2025.1442215)
Supplement: Supplementary file 2 [file Datasheet2.docx]

**ANNEX: Data Abstraction Tool**

1. **Participants identification**

Code No_________________Hospital_______________

Part I. Socio-demographic characteristics of ART experienced children and their family /caretaker:

1. Age in complete years--------------<1 year (months): -------------
2. Relationship of caregivers for the child:
3. Parent 2. Relatives 3. Guardians/neighbors
4. Age of the caregivers--------------
5. Family size------------------------
6. Monthly income of family in Ethiopian Birr---------------------------
7. Gender 1. Male 2. Female
8. Domicile 1. Urban 2. Rural
9. Religion 1. Orthodox 2. Muslim 3. Protestant 4. Catholic 5. Others
10. Ethnic group 1. Amhara 2. Tigray 3. Oromo 4. Others
11. Marital status of caregivers: 1. Single 2. Married 3. Divorced 4. Widowed 5. Separated
12. Educational status-- 1. No formal education 2. Elementary school

3. Secondary school 4. College and above

1. Occupation of caregivers: 1. Unemployed 2. Government employee 3. Farmer 4. House wife 5. Private employee 6. Daily laborer 7. Nongovernmental Organization employee 8. Self-employed
2. Parental status: 1. Both alive 2. Father alive 3. Mother alive 4. Both deceased 5. Unknown
3. HIV status of caretaker: 1. Positive 2. Negative 3. Not known
4. Number of months living with HIV----------------------------
5. Does anyone else know about your HIV Status (children and adolescents)? 1.Yes, 2. No
6. Family education: 1. No formal education 2. Primary school 3. Secondary school 4. College and above
7. Distance from home to ART clinic: 1. <10 Kilometers 2. >10 Kilometers

**Part II. Clinical characteristics:**

1. Recent CD4 count level (cells/µL) after using DTG-based regimen: --------------
2. Last virological outcomes before switching to DTG-based regimen: --------------
3. Recent virological outcomes after using DTG based regimen
4. Current WHO Clinical HIV/AIDS stage: 1. Stage I 2. Stage II, 3. Stage III, 4. Stage IV
5. Previous ART experience: 1. Naïve 2. Non-naive
6. If Non-naive mention last ART regimen before switch to DTG-based regimen:-----------------

1c, AZT‐3TC‐NVP 2. 1d, AZT‐3TC‐EFV 3. 1e, TDF‐3TC‐EFV 4.1f, TDF‐3TC‐NVP

1. Mention current ART regimen: 1. TDF+3TC+DTG 2. ABC+3TC+DTG 3. AZT+3TC+DTG 4. DRV/r+DTG+ABC+3TC 5. DRV/r+DTG+AZT+3TC 6. DRV/r+DTG+TDF+3TC
2. Virological Status prior initiation of DTG-based regimen.

1. Suppressed 2. Non-suppressed 3. Naïve

1. Duration on ART (in months): ----------------------------
2. Duration on DTG-ART (in months): ----------------------------
3. Hemoglobin: A. -------------------- B. Not done
4. History of isoniazid prophylaxis: 1. Yes 2. No
5. History of Cotrimoxazole prophylaxis: 1. Yes 2. No
6. Recent history of OIs: 1. Yes 2. No
7. If yes: 1. TB 2. Pneumonia 3. Oral thrush 4. Zoster 5. Diarrhea 6. CNS toxoplasmosis

7. Others mention it-------------------

1. History of sever acute malnutrition status: 1. No 2. Yes
2. Did you experience drug adverse effect/s after switching to DTG-based regimen? 1. Yes 2. No
3. How many ARV dose/s do you take/day? 1. Once 2. Twice
4. Did you miss ARV doses in the past one month? 1. Yes 2. No
5. If yes, how many dose/s did you miss? ---------------------
6. History of prevention of mother to child transmission (PMTCT) ARV services 1. Yes 2. No
